# Impact of anti-inflammatory drug consumption in peritonsillar abscesses: a retrospective cohort study

**DOI:** 10.1186/s12879-016-1761-2

**Published:** 2016-08-20

**Authors:** Thomas Feasson, Mathilde Debeaupte, Clément Bidet, Florence Ader, François Disant, Tristan Ferry, Christian Chidiac, Florent Valour

**Affiliations:** 1 General Medicine Department, Claude Bernard Lyon 1 University, Lyon, France; 2 Infectious Disease Department, Groupement Hospitalier Nord, Hôpital de la Croix-Rousse, Hospices Civils de Lyon, Lyon, France; 3 ENT Surgery Department, Groupement Hospitalier Est, Hôpital Edouard Herriot, Hospices Civils de Lyon, Lyon, France; 4 Department of Radiology, Centre Hospitalier de Valence, Valence, France; 5 Claude Bernard Lyon 1 University, Lyon, France; 6 INSERM U1111, International Center for Research in Infectiology (CIRI), Claude Bernard Lyon 1 University, Lyon, France

**Keywords:** Pharyngeal infection, Tonsillar abscess, Anti-inflammatory drugs, Nonsteroidal anti-inflammatory drugs, Corticosteroids

## Abstract

**Background:**

The experience of clinicians in charge of the in-hospital management of peritonsillar abscesses supports the association between severe forms and anti-inflammatory drug (AID) consumption. However, this observation is based on a limited number of clinical studies. Our objective was to assess the prevalence and impact of AID consumption in patients with peritonsillar abscesses.

**Methods:**

All patients referred to the ear, nose and throat surgery department for a peritonsillar abscess were included in a retrospective cohort study (2012–2014).

**Results:**

Among the 216 included patients (male, 55 %; median age, 32 years [IQR, 26–40]), 127 had received AID (59 %), including corticosteroids (*n* = 67, 31 %) and/or non-steroidal AIDs (NSAIDs, *n* = 76, 35 %). 199 patients (92 %) benefit from a puncture and 5 (2 %) from a surgery under general anesthesia, associated with ceftriaxone/metronidazole (51 %) or amoxicillin/clavulanic acid (46 %). An iterative surgical procedure was required in 93 cases (43 %), including 19 % under general anesthesia. Bacteriological analysis (79 %) mainly disclosed streptococci (66 %) of A (18 %) and/or milleri (33 %) groups. The prevalence of anaerobes was higher in patients using AIDs (46 % versus 29 %, *p* = 0.034), regardless of prior antibiotic therapy. 65 patients benefited from a CT-scan; AID consumption was associated with larger abscesses (6.8 [IQR, 3.7–12.7] versus 2.9 [IQR, 0.9–7.8] cm^3^; *p* = 0.005). AID consumption was not a risk factor of iterative surgical procedure.

**Conclusions:**

In comparison to the prescribing habits in uncomplicated upper respiratory tract infection, the high prevalence of AID consumption in patients with peritonsillar suppuration suggests a role of AIDs in promoting these complications.

## Background

Despite healthcare authorities’ warnings in the current guidelines about the use of corticosteroids and non-steroidal anti-inflammatory drugs (NSAIDs) in patients with upper respiratory tract infection, including pharyngitis, they are still widely/regularly used [1–4]. The experience of many physicians in charge of the in-hospital management of peritonsillar abscesses supports the association between anti-inflammatory drug (AID) consumption and such severe forms of infection. If the negative impact of long-term corticosteroid therapy has been well demonstrated in various clinical situations such as pyogenic infections, tuberculosis, severe forms of varicella, viral hepatitis B and C or invasive fungal infections [5–12], no study has linked the use of AIDs, including NSAIDs, with the occurrence of peritonsillar abscesses. In this setting, we aimed to evaluate the prevalence of AID consumption among patients with peritonsillar abscesses, and to evaluate the impact of AID on the presentation and management of these complications.

## Methods

### Study design

All patients referred to the ear, nose and throat (ENT) surgery department of our institution for peri-tonsillar abscess(es) between 1^st^ January 2012 and 31^st^ December 2014 were included in a retrospective single-center observational cohort. Patients were selected from a hospital information system determined by the International Classification of Diseases (ICD-10), 10^th^ revision, using the codes corresponding to peritonsillar abscess (J36), pharyngeal abscess (J39.0 and J39.1), cellulitis and abscess of the mouth (K12.2), cellulitis of the face (L03.2), cellulitis of other sites (L03.8) or unspecified cellulitis (L03.9) [13]. All medical records were reviewed to confirm that the final diagnosis corresponded to a peritonsillar abscess. Doubtful cases were validated or excluded independently by two of the study authors. In particular, patients with cervical suppurations with no clinical and radiological argument for a primitive tonsillar involvement have been excluded.

### Data collection

A standardized case report form was used to retrospectively collect demographic data (gender, age), major comorbidities (obesity, diabetes, immunosuppression, history of tonsil disease, and toxic habits), treatment received prior to hospital admission (antibiotics, NSAIDs and corticosteroids), biological inflammatory parameters at admission (C-reactive protein (CRP), white blood cell and neutrophil counts), results of microbiological analysis, surgical (puncture/surgery under general anesthesia (GA)) and medical management. When a CT-scan was performed, images were reviewed for abscesses’ volume measurement by a radiologist using the formula for approximation of the ellipsoid: 4/3 × π x ABC where A represents the largest diameter in the horizontal plan, and B and C the largest diameters in the two others orthogonal plans. All data were collected from the electronic medical records of the ENT and emergency departments and from the institutional software for biological results (CristalNet®).

### Statistical analysis

The usual methods of descriptive statistics were used to summarize the variables of the study, described by their size (n, %) for categorical variables and their median (interquartile range [IQR]) for continuous variables. The number of missing data was removed from the denominator for each percentage calculation. Non-parametric tests (Chi-2, Fisher exact test, Mann–Whitney U-test) were used to compare the study group, as appropriate. All analysis were performed using SPSS software (version 16.0; SPSS, Chicago, Illinois, USA).

## Results

### Included population

Two hundred and forty patients were admitted to the ENT surgery department of our institution for a suppuration of tonsil origin between January 1^st^ 2012 and December 31^st^ 2014. After exclusion of 24 patients for whom information regarding AID consumption was not available (10.0 %), 216 patients were finally enrolled in the study, including 119 men (55.1 %) with a median age of 32.5 years (IQR, 25.7–39.5). Main demographic characteristics and comorbidities of patients are summarized in Table 1.

**Table 1:** Demographics and comorbidities of the 216 included patients, and comparison according to the consumption of anti-inflammatory drugs prior to admission

	Total population	No AID consumption	AID consumption	*p*-value
Demographics	216	89 (41.2 %)	127 (58.7 %)	
Sex (male)	119 (55.1 %)	52 (58.4 %)	67 (52.8 %)	0.410
Age (years)	32.2 (25.7–39.5)	31.3 (25.6–38.9)	32.8 (25.7–39.7)	0.670
Comorbidities				
BMI (kg/cm^2^)	23.6 (21.7–26.5)	25.1 (22.0–28.6)	23.4 (20.6–25.8)	0.091
Diabetes	3 (1.4 %)	3 (3.4 %)	0 (0.0 %)	0.069
Chronic respiratory disease	2 (0.9 %)	1 (1.1 %)	1 (0.8 %)	1.000
Pharyngitis	24 (11.1 %)	13 (14.6 %)	11 (8.7 %)	0.171
Peritonsillar suppuration	16 (7.4 %)	9 (10.1 %)	7 (5.5 %)	0.204
Immunosuppression	5 (2.3 %)	1 (1.1 %)	4 (3.1 %)	0.651
Hematological malignancy or solid tumor	2 (0.9 %)	0 (0 %)	2 (1.6 %)	0.513
Tabaco consumption	63 (52.1 %)	30 (53.6 %)	33 (50.8 %)	0.758

Data are presented as n (%) for dichotomic variables and median (IQR) for continuous variables. For the calculation of each percentage, the number of missing values was excluded from the denominator. The two groups were compared by non-parametric tests (chi-square test, Fisher exact test and Mann–Whitney U-test), as appropriate

*AID* anti-inflammatory drug, *BMI* body mass index

### Pre-hospital treatment

One hundred and twenty-seven patients (58.4 %) had received AIDs prior to hospital admission, including 76 patients (59.8 %) receiving NSAIDs for a median duration of 3 days (IQR, 2.0–5.0) and 67 patients (52.8 %) treated by oral corticosteroids for 4 days (IQR, 2.0–6.0). Of note, 16 patients (7.4 %) received both NSAIDs and oral corticosteroids.

One hundred and thirty-six patients (63 %) had received antibiotics prior to hospital care. The main antibiotics prescribed before admission were amoxicillin and clavulanic acid (*n* = 56, 41.2 %), amoxicillin (*n* = 43, 31.6 %), macrolide (*n* = 17, 12.5 %), resulting in an effective anti-anaerobic therapy in 60 cases (44.1 %).

### In-hospital management

**Fig. 1:**
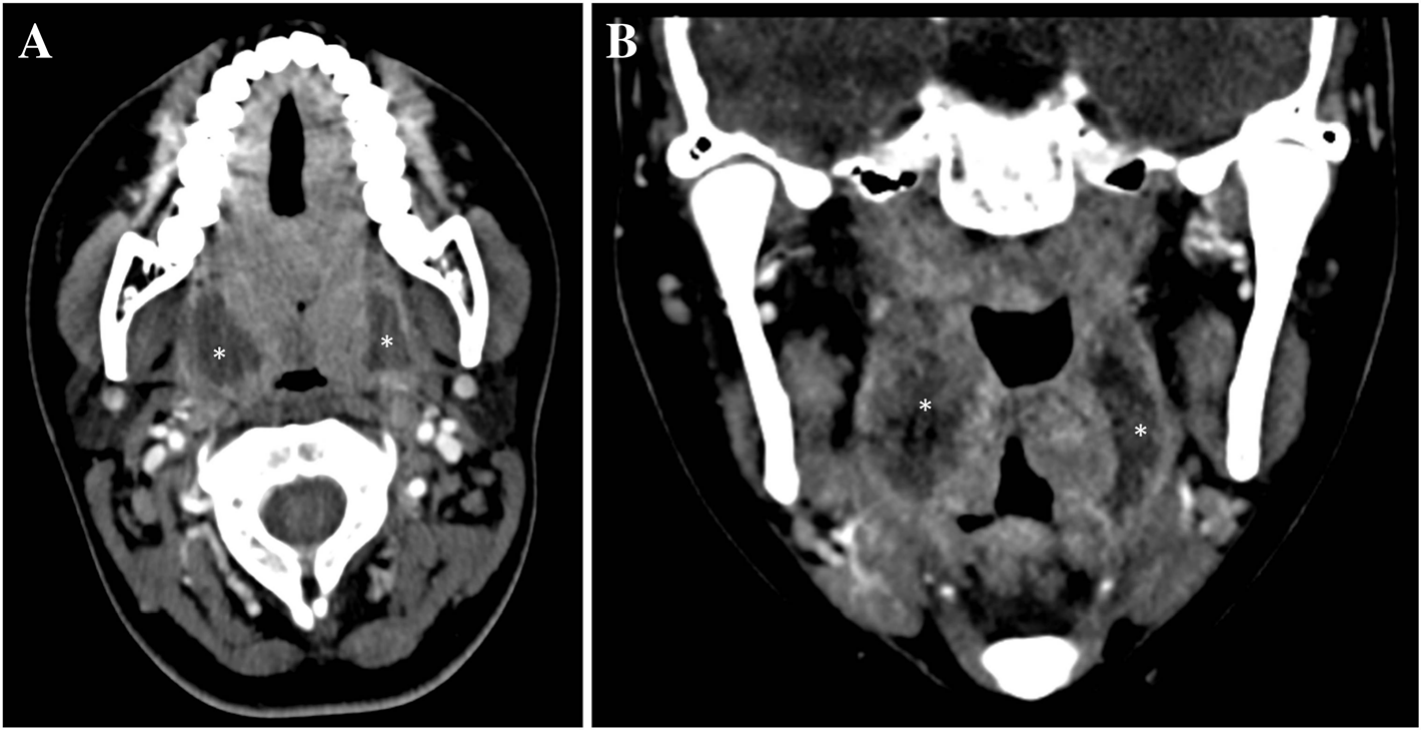
Horizontal (panel **a**) and coronal (panel **b**) CT-scan disclosing voluminous bilateral tonsillar abscesses (*asterisks*) in one of the patient included in the study who had consumed AID before hospital admission

All but one patient (99.5 %) were hospitalized for a median of 3 days (IQR, 3.0–4.0), 3 of whom required intensive care unit (ICU) admission. Upon admission, 77 patients (35.6 %) benefited from a CT scan, mainly in case of doubtful diagnosis. Images were available in 65 patients (30.1 %), of 6 (9.2 %) had bilateral abscesses (Fig. 1). The initial biological findings showed an inflammatory syndrome in all patients (i.e. CRP > 10 mg/L), with a median CRP level at 90.0 mg/L (IQR, 44.4–156.5). Two hundred and three patients (94 %) required an abscess drainage puncture upon admission, with or without incision under local anesthesia (LA) (*n* = 199, 92.1 %) or a surgical drainage/excision under general anesthesia (GA) (*n* = 5, 2.3 %). The incision under LA was deemed insufficient for one patient, requiring immediate surgical procedure under GA. Of note, the diagnosis of peritonsillar abscess was confirmed by CT-scan in 9 of the 13 patients who did not benefit from drainage. In the four others, the diagnosis was clinically based, even if the abscess did not appear voluminous enough to benefit from a puncture. Iterative surgical procedure was required in 93 patients (43.1 %), including 91 (97.8 %) transoral puncture/drainage (including 16 under GA) and two cervicotomies for local extension of the infection.

Two hundred and fourteen patients received intravenous antibiotics (99.1 %) for at least one day. The main antimicrobials were amoxicillin-clavulanic acid (*n* = 98, 45.4 %) or ceftriaxone-metronidazole combination (*n* = 110, 51 %), except in rare cases of allergy. The total duration of antibiotic therapy was 12.5 days (IQR, 11.0–15.0), including 10.0 days (IQR, 10.0–11.0) from hospital admission.

No descending mediastinitis was observed. No related-fatality case was recorded.

### Bacteriological findings

Microbiological analysis were performed in 169 (78.2 %) patients, yielding a plurimicrobial infection in 36.1 % of cases (*n* = 61). The most frequently involved bacteria were streptococci of milleri group (32.7 %) followed by *Fusobacterium* spp. (25.6 %; predominantly including *F. necrophorum* [*n* = 30, 90.7 %]) and group A *Streptococcus* (17.9 %). All of the bacteriological results are presented in Table 2.

**Table 2:** Bacteriological findings in 169 of the 216 patients included, and comparison according to the consumption of anti-inflammatory drugs prior to admission

	Total population	No AID	AID	AID vs. no AID	NSAID	NSAID vs. no AID^a^	Corticosteroids	Corticosteroids vs. no AID^a^	NAIDS vs. corticosteroids^a^
				*p*-value		*p*-value		*p*-value	*p*-value
Bacteriological analysis	169 (78.2 %)	65 (73.0 %)	104 (81.9 %)	0.125	65 (85.5 %)	0.268	55 (82.1 %)	0.588	0.632
*Streptococcus* spp	110 (65.5 %)	44 (67.7 %)	66 (64.1 %)	0.631	40 (62.5 %)	0.424	37 (67.3 %)	0.914	0.548
*S. pyogenes*	30 (17.9 %)	17 (26.2 %)	13 (12.6 %)	0.026	10 (15.6 %)	0.512	3 (5.5 %)	0.022	0.131
*S. milleri group*	55 (32.7 %)	19 (29.2 %)	36 (35.0 %)	0.442	19 (29.7 %)	0.452	25 (45.5 %)	0.136	0.040
*Staphylococcus aureus*	6 (3.6 %)	4 (6.2 %)	2 (1.9 %)	0.207	1 (1.6 %)	0.393	1 (1.8 %)	0.648	1.000
*Haemophilus* spp	4 (2.4 %)	2 (3.1 %)	2 (1.9 %)	0.641	1 (1.6 %)	1.000	1 (1.8 %)	1.000	1.000
Anaerobes	66 (39.3 %)	19 (29.2 %)	47 (45.6 %)	0.034	26 (40.6 %)	0.070	25 (45.5 %)	0.012	0.457
*Fusobacterium* spp	43 (25.6 %)	11 (16.9 %)	32 (31.1 %)	0.041	18 (28.1 %)	0.043	16 (29.1 %)	0.028	0.802
*Prevotella* spp	11 (6.5 %)	2 (3.1 %)	9 (8.7 %)	0.206	6 (9.4 %)	0.110	4 (7.3 %)	0.361	0.726
Plurimicrobial infection	61 (36.1 %)	20 (30.8 %)	41 (32.5 %)	0.254	24 (36.9 %)	0.658	24 (43.6 %)	0.186	0.395

Data are presented as n (%). For the calculation of each percentage, the number of missing values was excluded from the denominator. Groups were compared by non-parametric tests (chi-square and Fisher exact tests), as appropriate

*AID* anti-inflammatory drug, *NSAID* non-steroidal anti-inflammatory drug, *vs.* versus

^a^Excluding patients receiving both NSAIDs and corticosteroids

### Impact of anti-inflammatory drugs

The demographic characteristics of patients receiving or not AID prior to admission were similar (Table 1). Pre-hospital antibiotic use was more frequent in patients receiving AID (70.1 % versus 52.8 %; *p* = 0.010). AID consumption did not impact hospital length of stay (median of 3.0 days for both groups; *p* = 0.17) nor ICU admission (2 in the AID group and 1 in the group without AID). Baseline CRP level was significantly higher in the group without AID (109.5 mg/L [IQR, 66.9–172.5] versus 72.4 mg/L [IQR, 38.3–133]; *p* = 0.002). Conversely, white blood cell count was higher in patients treated with AID (15,400 cells/mm^3^ [IQR, 12,500–18,300] versus 13,500 cells/mm^3^ [IQR, 12,000–16,200]; *p* = 0.031). Concerning radiological results, 6 patients had bilateral abscesses, including 4 (11.4 %) in the AID group and 2 (6.7 %) in patients without AID (*p* = 0.678). AID consumption was associated with significantly larger abscesses (6.8 [IQR, 3.7–12.7] versus 2.9 [IQR, 0.9–7.8] cm^3^; *p* = 0.005). Regarding bacteriological findings, the prevalence of anaerobic bacteria was higher in patients who received AIDs (45.6 % versus 29.2 %; *p* = 0.034), through an overrepresentation of *Fusobacterium* (31.1 % versus 16.9 %; *p* = 0.041). Given these results, additional analysis comparing patients infected or not by anaerobes showed no significant difference regarding the nature of antimicrobial use before sampling (Table 3).

**Table 3:** Comparison of pre-hospital antibimicrobial use in patients infected or not by anaerobic bacteria

	Anaerobes	No anaerobes	*p*-value
n	66	102	
Pre-hospital antibiotic therapy	42 (63.6 %)	64 (62.7 %)	0.907
Amoxicillin	10 (15.2 %)	23 (22.5 %)	0.239
Amoxicillin - clavulanic acid	15 (22.7 %)	28 (27.5 %)	0.493
Clindamycin	2 (3.0 %)	0 (0.0 %)	0.153
Anti-anaerobes therapy	17 (28.5 %)	29 (28.4 %)	0.704
Antimicrobial therapy duration (days)	4.0 (3.0–7.0)	3.0 (2.0–5.0)	0.265

Data are presented as n (%) for dichotomic variables and median (IQR) for continuous variables. For the calculation of each percentage, the number of missing values was excluded from the denominator. The two groups were compared by non-parametric tests (chi-square test, Fisher exact test and Mann–Whitney U-test), as appropriate

Regarding in-hospital management, there was no difference between the needs for surgery under GA and/or iterative drainages between the two groups (Table 4). However, the puncture was more frequently successful in draining pus in patients treated by AIDs (75.6 % versus 62.9 %; *p* = 0.045). Of note, two patients required cervicotomy, all in the AID group (1.6 %). AID consumption impacted neither the nature nor the duration of antibiotic use.

**Table 4:** In-hospital management of the 216 included patients, and comparison according to the consumption of anti-inflammatory drugs prior to admission

	Total population	No AID	AID	AID vs. no AID	NSAID	NAID vs. no AID^a^	CT	Corticosteroids vs. no AID^a^	NSAID vs. corticosteroids^a^
				*p*-value		*p*-value		*p*-value	*p*-value
n	216	89 (41.2 %)	127 (58.7 %)		76 (35.2 %)		67 (31.0 %)		
Paraclinical tests									
CT-scan	77 (35.6 %)	38 (42.7 %)	39 (30.7 %)	0.070	23 (30.3 %)	0.305	19 (28.4 %)	0.185	0.842
Abscess volume (cm^3^)	4.4 (1.6–10.2)	2.9 (0.9–7.8)	6.8 (3.7–12.7)	0.005	5.7 (3.2–10.6)	0.028	7.1 (1.9–13.5)	0.049	0.728
CRP (mg/L)	90.0 (44.4–156.5)	109.5 (66.9–172.8)	72.4 (38.3–133.0)	0.002	95.1 (50.9–181.0)	0.663	47.5 (26.5–81.5)	<10^−3^	<10^−3^
WBC (/mm^3^)	14,400 (12,100–17,700)	13,500 (12,000–16,200)	15,400 (12,50–18,300)	0.031	15,600 (12,600–17,700)	0.131	15,500 (12,500–19,100)	0.111	0.605
Neutrophils (/mm^3^)	13,600 (10,100–16,200)	12,800 (9400–15,600)	14,100 (10,400–16,500)	0.326	14,200 (11,300–15,600)	0.437	14,100 (10,300–17,000)	0.454	0.927
Hospitalisation	215 (99.5 %)	88 (98.9 %)	127 (100.0 %)	0.412	76 (100.0 %)	1.000	67 (100.0 %)	1.000	NC
Hospital stay (d)	3.0 (3.0–4.0)	3.0 (3.0–4.0)	3.0 (3.0–4.0)	0.170	3.0 (3.0–4.0)	0.492	3.0 (3.0–4.0)	0.066	0.344
ICU	3 (1.4 %)	1 (1.1 %)	2 (1.6 %)	1.000	2 (2.6 %)	0.565	0 (0.0 %)	1.000	0.499
Surgical management	203 (94 %)	83 (93.3 %)	120 (94.5 %)	0.708	73 (96.1 %)	0.740	63 (94 %)	1.000	0.539
Puncture/Incision	199 (92.1 %)	82 (92.1 %)	117 (92.1 %)	0.998	71 (93.4 %)	0.918	62 (92.5 %)	0.693	0.787
Productive puncture	152 (70.4 %)	56 (62.9 %)	96 (75.6 %)	0.045	58 (76.3 %)	0.268	53 (79.1 %)	0.160	0.831
Initial	199 (92.1 %)	82 (92.1 %)	117 (92.1 %)	0.998	71 (93.4 %)	0.918	62 (92.5 %)	0.758	0.787
Secondary	80 (37 %)	34 (38.2 %)	46 (36.2 %)	0.767	31 (40.8 %)	0.849	24 (35.8)	0.294	0.419
Surgery under GA	21 (9.7 %)	9 (10.1 %)	12 (9.4 %)	0.871	10 (13.2 %)	0.982	6 (9.0 %)	0.328	0.217
Initial	5 (2.3 %)	1 (1.1 %)	4 (3.1 %)	0.330	3 (3.9 %)	0.346	2 (3.0 %)	1.000	1.000
Secondary	18 (8.3 %)	8 (9.0 %)	10 (7.9 %)	0.806	9 (11.8 %)	0.836	4 (6.0 %)	0.155	0.122
Cervicotomy	2 (0.9 %)	0 (0.0 %)	2 (1.6 %)	0.513	2 (2.6 %)	0.161	0 (0.0 %)	NC	0.499
Tonsillectomy	12 (5.6 %)	4 (4.5 %)	8 (6.3 %)	0.765	7 (9.2 %)	0.715	4 (6.0 %)	0.653	0.372
Iterative procedure	93 (43.1 %)	39 (43.8 %)	54 (42.5 %)	0.849	38 (50.0 %)	0.887	27 (40.3 %)	0.147	0.142
Medical management									
IV antimicrobial therapy	214 (99.1 %)	88 (98.9 %)	126 (99.2 %)	1.000	75 (98.7 %)	0.410	66 (98.5 %)	1.000	NC
Total duration (d)	12.5 (11.0–15.0)	12.0 (11.0–15.0)	13.0 (10.0–16.0)	0.249	12.0 (10.0–15.3)	0.203	14.0 (12.0–17.5)	0.046	0.007
From hospital admission (d)	10.0 (10.0–11.0)	10.0 (10.0–12.0)	10.0 (10.0–11.0)	0.021	10.0 (10.0–11.0)	0.204	10.0 (10.0–11.0)	0.008	0.176

Data are presented as n (%) for dichotomic variables and median (IQR) for continuous variables. For the calculation of each percentage, the number of missing values was excluded from the denominator. The two groups were compared by non-parametric tests (chi-square test, Fisher exact test and Mann–Whitney U-test), as appropriate

*AID* anti-inflammatory drug, *CRP* C-reactive protein, CT-scan, computed tomography scan, *d* days, *GA* general anesthesia, *ICU* Intensive care unit, *IV* intravenous, *NSAID* non-steroidal anti-inflammatory drug, *WBC* white blood cell

^a^Excluding patients receiving both NSAIDs and CT

Finally, no differences were found between patients receiving NSAIDs or corticosteroids.

## Discussion

This study reports the largest series documenting the impact of AIDs in patients with peritonsillar abscesses. Despite limitations related to its retrospective and observational nature, the proportion of patients excluded because of missing data regarding the primary outcome (AID consumption) was low (10 %), thanks to the precision of the medical records, thus limiting a possible selection bias.

The first important result is the high frequency of AIDs pre-hospital use in patients admitted in an ENT surgery department for peritonsillar suppuration, compared to the known prescription habits in tonsillitis and upper respiratory infections. According to a 2013 French survey, 46 % of general practitioners (GPs) are prescribers of NSAIDs in upper respiratory tract infections [3]. In another study investigating GP and pediatrician prescription in 701 adults and 758 children treated for upper respiratory tract infection, NSAIDs and/or oral corticosteroids were prescribed in 9 to 23 % of all cases, and in 15 to 22 % of tonsillitis [4]. The few available data regarding self-medication in this setting reveals an AID consumption rate of 14 % in sore throat [14]. In comparison, the prevalence approaching 60 % of AIDs consumption in patients presenting a complicated infection suggests a role of corticosteroids and NSAIDs in the genesis and evolution of peritonsillar suppuration. However, a comparative study of patients with upper respiratory tract infection consuming or not AIDs is required to definitively draw this conclusion.

The AID impact on the inflammation process has been well described, resulting in the inhibition of phagocytosis [15, 16]. Corticosteroids inhibit the production and secretion of proinflammatory cytokines by all immune cells, and increase the number of neutrophils by reducing their adhesion to the vascular endothelium, thus preventing their diapedesis to the infection site [17]. NSAIDs impede prostaglandin production by blocking the action of cyclooxygenase [18]. In addition to their intended analgesic effect, NSAIDs inhibit all stages of the innate and acquired immune responses by i) hindering several membrane enzymes of neutrophils, macrophages and platelets, thus preventing their migration and chemotaxis [19]; ii) stabilizing lysosomal membranes, therefore limiting degranulation [20]; iii) inhibiting antibody production in human B-cells [21]; and iv) suppressing the proliferation of CD4+ and CD8+ T-cells [22]. The high white blood cell count and low CRP level observed in the AID group of patients of our study are the resultant of all these mechanisms, and suggest that the doses of AID consumed by the included patients, even for a short period, had an effect on the inflammatory processes.

The clinical consequences of the immunosuppression state induced by long-term corticosteroid therapies have been highlighted in various clinical situations [5–12]. The role of NSAIDs in the development and/or worsening of infections is less clear, but strongly suspected. For example, NSAIDs increase the risk of skin and soft tissue bacterial infection when used during chickenpox [23, 24]. Their involvement in the pathogenesis of severe fasciitis and cellulitis has also been suggested by several studies [25–29]. They have been suspected to increase the frequency of pleural effusions, to lengthen the duration of oxygen therapy and to major ICU admission in patients hospitalized for community-acquired pneumonia [30]. Finally, NSAIDs increase the risk of complications in acute pyelonephritis [31]. However, no similar studies exist in upper respiratory tract infections, despite the use of AID is suspected of being partly responsible for the recent increased incidence of peritonsillar abscesses [32]. Indeed, Demeslay et al. showed that 60 % of 163 patients suffering from peritonsillar abscess or cervical cellulitis had taken AIDs [33]. In another study by Lepelletier et al., a relation between the occurrence of a peritonsillar abscess and self-medication with AIDs has also been suggested, finding a 65 % exposure to AIDs before the onset of this complicated infection [14]. Finally, Thiebaut et al. observed a high rate of AID cunsumption in patients with cervical cellulitis with or without descending mediastinitis [34].

In our study, patients using or not AIDs had similar demographics and comorbidities, particularly with respect to known risk factors for peritonsillar cellulitis as male gender, tobacco use, tonsillitis history and immunocompromised status. No difference regarding in-hospital surgical or medical management was observed. However, abscesses were more than two and a half larger in patients consuming AID, and the two patients requiring cervicotomy had received NSAIDs. If the correlation between pre-hospital AID and antibiotic uses cannot rule out the existence of a more severe disease on first presentation in patients receiving AID, it seems more likely that GPs were reluctant to prescribe AID without antibiotics in a septic context.

Finally, bacteriological findings were consistent with the literature data, dominated by oral flora streptococci of milleri group, *Fusobacterium* and *Streptococcus pyogenes* [35]. An interesting feature was the observation of a higher proportion of anaerobes in the AID group, unrelated with the antibiotic therapy received before sampling. No similar finding has been reported to date. No explanation can be advanced with certainty, but these results suggest an influence of AIDs on the immune system facilitating anaerobe infections. The impact of AIDs on the pharyngeal microbiota may also be evaluated. Another explanation may lies in a possible AID-related high inoculum effect, facilitating the detection of fastidious organisms such as anaerobes, as supported by the more frequently successful puncture and the larger abscess size observed on CT-scan in patient receiving AIDs.

## Conclusions

In conclusion, the high prevalence (approaching 60 %) of AID consumption among patients with peritonsillar abscess suggests a role of these drugs in the development of severe complications of common infections. Associated with their only symptomatic benefit, this observation encourages interest in renewing a cautious attitude about their systematic use in infectious diseases. However, larger prospective studies are required to definitely establish whether AID consumption constitute a trigger for severe complication during upper respiratory tract infection. Moreover, AID consumption was associated with significantly larger abscesses, and with a higher prevalence of anaerobes, justifying the combination of ceftriaxone and metronidazole as first-line treatment.

## Abbreviations

AID, anti-inflammatory drug; BMI, body mass index; CRP, C-reactive protein; CT, computed tomography; d, day; ENT, ear, nose and throat; GA, general anaesthesia; GP, general practitioner; ICD, international classification of diseases; ICU, intensive care unit; IQR, interquartile range; IV, intravenous; LA, local anaesthesia; NSAID, non-steroidal anti-inflammatory drug; vs, versus; WBC, white blood cell
